# Clinical course and outcomes of diagnosing Inflammatory Bowel Disease in children 10 years and under: retrospective cohort study from two tertiary centres in the United Kingdom and in Italy

**DOI:** 10.1186/s12876-016-0455-y

**Published:** 2016-03-15

**Authors:** Marco Gasparetto, Graziella Guariso, Laura Visona’ Dalla Pozza, Alexander Ross, Robert Heuschkel, Matthias Zilbauer

**Affiliations:** Cambridge University Hospitals, Addenbrooke’s, Department of Paediatric Gastroenterology, Hepatology and Nutrition, Box 116 Level 8, Cambridge Biomedical Campus, Hills Road, Cambridge, CB2 0QQ UK; Padova University Hospital, Department of Women and Children’s Health, Unit of Paediatric Gastroenterology, Padova, 35128 Italy; Padova University Hospital, Department of Women and Children’s Health, Unit of Epidemiology and Community Medicine, Padova, 35128 Italy

**Keywords:** Inflammatory Bowel Disease (IBD), Age, Children, Presentation, Location, Severity, Outcomes

## Abstract

**Background:**

Most children with Inflammatory Bowel Disease (IBD) are diagnosed between 11 and 16 years of age, commonly presenting with features of typical IBD. Children with onset of gut inflammation under 5 years of age often have a different underlying pathophysiology, one that is genetically and phenotypically distinct from other children with IBD. We therefore set out to assess whether children diagnosed after the age of 5 years, but before the age of 11, have a different clinical presentation and outcome when compared to those presenting later.

**Methods:**

Retrospective cohort study conducted at two European Paediatric Gastroenterology Units. Two cohorts of children with IBD (total number = 160) were compared: 80 children diagnosed between 5 and 10 years (Group A), versus 80 children diagnosed between 11 and 16 (Group B). Statistical analysis included multiple logistic regression.

**Results:**

Group A presented with a greater disease activity (*p* = 0.05 for Crohn’s disease (CD), *p* = 0.03 for Ulcerative Colitis (UC); Odds Ratio 1.09, 95 % Confidence Interval: 1.02–1.1), and disease extent (L2 location more frequent amongst Group A children with CD (*p* = 0.05)). No significant differences were observed between age groups in terms of gastro-intestinal and extra-intestinal signs and symptoms at disease presentation, nor was there a difference in the number of hospitalisations due to relapsing IBD during follow-up. However, children in Group A were treated earlier with immunosuppressants and had more frequent endoscopic assessments.

**Conclusion:**

While clinicians feel children between 5 and 10 years of age have a more severe disease course than adolescents, our analysis also suggests a greater disease burden in this age group. Nevertheless, randomized trials to document longer-term clinical outcomes are urgently needed, in order to address the question whether a younger age at disease onset should prompt *per se* a more “aggressive” treatment. We speculate that non-clinical factors (e.g. genetics, epigenetics) may have more potential to predict longer term outcome than simple clinical measures such as age at diagnosis.

**Electronic supplementary material:**

The online version of this article (doi:10.1186/s12876-016-0455-y) contains supplementary material, which is available to authorized users.

## Background

The incidence of Inflammatory Bowel Disease (IBD) is rising worldwide, with a current mean prevalence in the general population estimated at 1/1,000 inhabitants in Europe and North America, and a rising trend observed in developing countries [1, 2]. Although patients may develop IBD at any age, currently approximately 10–25 % of patients are diagnosed during childhood or adolescence [3–7]. The majority of children diagnosed with IBD will present between 11 and 16 years of age. However, in recent years the reports of children diagnosed below the age of 10 years seems to be steadily increasing [8–12]. While a more aggressive presentation and disease behaviour has been well established for children diagnosed under the age of 5 years (i.e. early onset and very early onset IBD) [13–18], little is currently known about specific differences in phenotype and disease outcome for children diagnosed between 5 and 10 years of age. Previous reports have indicated that the age of disease onset correlates inversely with disease outcome, suggesting that younger age, even amongst children, may represent an important risk factor for an aggressive, treatment-resistant disease phenotype [19–24].

A so-called ‘step-up’ treatment approach remains standard practice in most European centres, which, at least in part, is due to the lack of validated prognostic markers that can identify high risk individuals. There are no studies that identify any aspects of disease phenotype that accurately predict disease course and outcome for an individual patient.

However, more recent evidence suggests that earlier use of more effective treatments (i.e. ‘top-down’ approach) in patients improves long-term outcome [25].

In this study, we therefore aimed to assess whether children diagnosed with IBD between their 5^th^ and 11^th^ birthday differ significantly in disease phenotype, course and response to treatment, when compared to children diagnosed in later childhood.

We compared two groups of children diagnosed between 5–10 and 11–16 years, recruited at two European paediatric gastroenterology centres.

## Methods

### Study population

This retrospective cohort study was conducted at two tertiary Paediatric Gastroenterology Units, in Cambridge - United Kingdom, and in Padua - Italy. Patients enrolled were all diagnosed between 2009 and 2013 to ensure a minimum follow-up of 12 months.

Ethical approval for the study was obtained from both Institutions (Cambridge University Hospitals NHS FT, Department of Paediatric Gastroenterology, IBD Internal Audit - Cambridge, UK; Medical Ethics Committee at the Department of Women’s and Children’s Health - Padua University, Padova, Italy). Each patient recruited and their parents/guardians were given appropriate information for consent.

A total of 287 patients from the two countries were eligible for the study. Plotting the distribution of their age at diagnosis of IBD, we also identified 11 years as the value separating the lowest quartile of children (5–10 yeras) from the rest (11–16 yeras). Hence, this age cut-off was chosen as a suitable watershed between the two age groups, as it also represents a plausible boundary line between childhood and adolescence, and as various age cut-offs (i.e. 10,11 and 12 years of age) have been used in previous descriptive studies [7–9, 24].

We therefore selected children diagnosed between 5 and 10 years of age (Group A) and those diagnosed between 11 and 16 (Group B).

A total of 160 patients were selected from children in each service as this number was available in each unit and age-group. Patients were grouped as follows: 80 children in Group A (40 British and 40 Italians), and 80 children in Group B (40 British and 40 Italians) (Table 1).

**Table 1:** Distribution of the patients enrolled, grouped by age at diagnosis (group A, 5 to 10 years, versus group B, 11 to 16 years of age at diagnosis), by type of Inflammatory Bowel Disease, gender and ethnicity. Disease location, CD behaviour, Growth (for CD) and Severity (for UC) are also displayed according to the Paris Classification [28]

Age at diagnosis	Brit^a^ Group A (5 to 10 years)	Brit Group B (11–16 years)	Ita^b^ Group A (5 to 10 years)	Ita Group B (11–16 years)
Patients’ number	40	40	40	40
Distribution by gender	23 M^c^ 17 F^d^	25 M 15 F	15 M 25 F	20 M 20 F
Age at diagnosis				
Mean ± SD^e^	7.65 ± 2.38	14.1 ± 1.03	7.4 ± 3.1	13.4 ± 1.39
Median	8.34	14.3	7.9	13.5
Range	5.1–10.6	11.4–16.6	5.6–10.8	11.2–16.3
Distribution by type of IBD^f^	15 CD^g^	19 CD	16 CD	22 CD
	4 IBD-U^h^ CD-like	5 IBD-U CD-like	1 IBD-U CD-like	1 IBD-U CD-like
	12 UC^i^	7 UC	15 UC	15 UC
	9 IBD-U UC-like	9 IBD-U UC-like	8 IBD-U UC-like	2 IBD-U UC-like
Follow-up duration (years)				
Mean ± SD	3.1 ± 2.3	1.92 ± 1.19	4.5 ± 3.71	2.21 ± 2.4
Median	2.8	1.72	4.2	1.17
Range	0.1–11.14	0.3–4.62	0.04–11.7	0.07–9.5
Ethnicity	36 white British	38 white British	34 white Italians	38 white Italians
	3 white non-British (Belgium, Italy, Romania)	1 white non British (Turkey)	6 white non-Italians (2 from Romania, 2 from Moldova, 1 from Kosowo, 1 Gipsy)	1 white non-Italian (Romania)
	1 African (Morocco)	1 African (Egypt)		1 African (Morocco)
Disease location (Paris Classification [28])	CD	CD	CD	CD
	2 L1 + L4a	1 L1/2 L1 + L4a/1 L1 + L4b	1 L1	2 L1/2 L1 + L4a
	2 L2/4 L2 + L4a	1 L2 + L4a	5 L2/1 L2 + L4a	1 L2/1 L2 + L4a
	9 L3 + L4a/2 L3 + L4b	3 L3/16 L3 + L4a	3 L3/7 L3 + L4a	10 L3/7 L3 + L4a
	UC	UC	UC	UC
	1 E1/2 E2	1 E1/2 E2	1 E1/7 E2	2 E1/4 E2
	1 E3/17E4	2 E3/11 E4	1 E3/14 E4	2 E3/9 E4
CD behaviour (Paris Classification [28])	11 B1, 5 pB1	11 B1, 4 pB1	10 B1, 2 pB1	5 B1, 5pB1
	1 B2	5 B2	1 B2, 1 pB2	10 B2, 1 pB2
	1B3	1 B3, 1 pB3	1pB3	1 pB3
	1pB2B3	1 B2B3, 1 pB2B3	1 B2B3, 1 pB2B3	1 B2B3
CD growth (Paris Classification [28])	4 S1	3 S1	3 S1	2 S1
	17 S0	13 S0	20 S0	15 S0

^a^ *Brit* British

^b^ *Ita* Italian

^c^ *M* male

^d^ *F* female

^e^ *SD* standard deviation

^f^ *IBD* inflammatory bowel disease

^g^ *CD* Crohn’s disease

^h^ *IBD-U* inflammatory bowel disease unclassified

^i^ *UC* ulcerative colitis

The groups from the United Kingdom (UK) and from Italy were comparable in age of disease onset: 7.65 ± 2.38 (mean age (years) ± standard deviation) for British Group A v. 7.4 ± 3.1 for Italian Group A; 14.1 ± 1.03 for British Group-B v. 13.4 ± 1.39 for Italian Group B (Further demographic data on Table 1).

### Data collection, clinical phenotype and outcome measurement

Clinical and outcome data were collected from patients’ records (paper notes and electronic databases). The same researcher (MG) was solely and directly involved in data collection. He was employed as a trainee doctor in both centres for longer than 1 year in each Institution. Therefore, the data collection was homogenous and was conducted with full awareness of the similarities and dissimilarities in protocols and practice between the two centres.

An Excel database was set up and appropriate software packages (including Graph Pad Prism version 6.4 and SAS statistical package version 9.4) were used for further statistical analysis of data.

In order to compare the children in Group A to the ones in Group B, a number of clinical variables were agreed *a priori* for collection from each patient in the four subgroups.

To depict the characteristics of the disease at diagnosis, we considered all clinical and biochemical variables included in the Paediatric Crohn’s Disease Activity Index (PCDAI) [26] and in the Paediatric Ulcerative Colitis Activity Index (PUCAI) [27]. Disease location was reported according to the Paris classification, basing on endoscopic findings [28].

The clinical variables collected for each patient at diagnosis, were the following:

- Abdominal pain: none, mild (can be ignored), severe (cannot be ignored);
- Stool consistency of most stools: formed, partially formed, completely unformed;
- Number of stools per 24 h: 0–2, 3–5, 6–8, >8;
- Rectal bleeding: none, small amount only - in less than 50 % of stools, small amount with most stools, large amount (50 % of the stool content);
- Nocturnal stools (any episode causing wakening);
- Patient functioning - general well-being within the last week: no limitation of activities - well, occasional difficulties in maintaining age appropriate activities - below par, frequent limitation of activities - very poor;
- Weight: weight gain or voluntary weight loss, involuntary weight loss 1–9 %, weight loss >10 %;
- Height: < 1 channel decrease (or height velocity > − SD), > 1 <2 channel decrease (or height velocity < − 1 SD > − 2 SD), >2 channel decrease (or height velocity < −2 SD);
- Abdomen on examination: no tenderness - no mass, tenderness - or mass without tenderness, tenderness - involuntary guarding - definite mass;
- Peri-rectal disease: none - asymptomatic tags, 1–2 indolent fistula - scant drainage - tenderness of abscess, active fistula - drainage - tenderness or abscess;
- Extra-intestinal manifestations including fever >38.5 for 3 days in a week, arthritis, uveitis, erythema nodosum, pyoderma gangrenosum: none, one, two;

Amongst the clinical variables at diagnosis, we also recorded urgency - tenesmus, nausea - vomit, mouth ulcers and iron-deficiency anaemia.

The statistical analyses, aimed at comparing the two age groups, were done on all IBD patients first, and subsequently on subgroups by diagnosis (CD and UC) (Table 2, Additional file 1: Table S1). Patients from the two centres were first grouped and analysed separately; only as part of a second step were they pooled together in order to compare the two age groups irrespective of the country of origin. The multiple logistic regression analysis was first performed including the country of origin as an independent variable, with repeated analysis then disregarding this variable.

**Table 2:** Comparison between children in group A (age at diagnosis of Inflammatory Bowel Disease between 5 and 10 years) and those in group B (age at diagnosis of Inflammatory Bowel Disease between 11 and 16 years): disease activity and location at presentation (A), biochemical parameters at disease presentation (B), disease course and outcomes (C)

Variables	All Group A vs Group B	Brit^a^ Group A vs Group B	Ita^b^ Group A vs Group B
A. Disease activity and location at presentation			
Disease activity			
CD^c^ (PCDAI^d^)			
Group A: mean ± SD^e^	38.75 ± 2.7	45 ± 3.3	31.3 ± 3.44
Group B:mean ± SD	30.76 ± 2.02	32.2 ± 2.26	29.6 ± 3.5
*P*	***0.05***	***0.003***	*0.7*
UC^f^ (PUCAI^g^)			
Group A: mean ± SD	47.2 ± 2.5	55.2 ± 2.2	41.2 ± 2.7
Group B: mean ± SD	41.4 ± 1.8	41.6 ± 2.5	39.8 ± 3.65
*P*	***0.03***	***0.03***	*0.8*
OR^h^	**1.09**	**1.11**	1.03
95 % CI^i^	**1.02–1.1**	**1.03–1.2**	1.01–1.08
Disease location			
CD			
*P*	*0.3* for L1	*0.6* for L1	*0.3* for L1
	***0.05*** **for L2**	***0.04*** **for L2**	*0.7* for L2
	*0.4* for L3	*0.5* for L3	*0.7* for L3
UC			
*P*	*0.7* for E1–E2	*0.8* for E1–E2	*0.6* for E1–E2
	*0.8* for E3–E4	*0.9* for E3–E4	*0.9* for E3–E4
B. Biochemical parameters at disease presentation			
WBC^j^ (x10^3/mm3)			
Group A: mean ± SD	10.1 ± 0.45	10.9 ± 0.7	9.4 ± 0.6
Group B: mean ± SD	8.81 ± 0.35	9.09 ± 0.5	8.5 ± 0.47
*P*	***0.03***	***0.04***	*0.3*
HCT^k^ (%)			
Group A: mean ± SD	33.7 ± 0.57	32.6 ± 0.6	34.8 ± 0.9
Group B: mean ± SD	35.8 ± 0.5	34.9 ± 0.7	36.7 ± 0.6
*P*	***0.005***	***0.02***	*0.09*
Platelet count (x10^3/mm3)			
Group A: mean ± SD	487 ± 18.9	476 ± 21.3	497.5 ± 31.2
Group B: mean ± SD	393 ± 13.4	404 ± 18.6	382 ± 19.3
*P*	***0.002***	***0.01***	***0.003***
C. Disease course and outcomes			
Early treatment with thiopurines (within 3 months of diagnosis)			
Group A	58	20	38
Group B	46	17	29
*P*	***0.05***	*0.7*	***0.006***
OR	**1.86**	1.04	**7.19**
95 % CI	1.02–4.33	0.8–2.63	0.9–77.4
Relapses (per patient/per year of follow-up)			
Group A: mean ± SD	1.4 ± 0.2	1.53 ± 1.24	1.03 ± 0.3
Group B: mean ± SD	0.85 ± 0.1	0.9 ± 0.7	0.7 ± 0.13
*P*	***0.05***	***0.005***	*0.3*
OR	**1.2**	**1.5**	**1.2**
95 % CI	**1.01–1.65**	0.8–4.45	0.74–2.64
Number of endoscopies (per patient/per year of follow-up)			
Group A: mean ± SD	0.9 ± 0.05	0.9 ± 0.06	0.9 ± 0.09
Group B: mean ± SD	0.75 ± 0.05	0.8 ± 0.07	0.7 ± 0.08
*P*	***0.04***	*0.2*	***0.05***
OR	**1.67**	**1.2**	**7.07**
95 % CI	**1.01–4.19**	0.98–1.58	**1.09–45.9**

^a^ *Brit* British

^b^ *Ita* Italian

^c^ *CD* Crohn’s disease

^d^ *PCDAI* paediatric Crohn’s disease activity index

^e^ *SD* standard deviation

^f^ *UC* ulcerative colitis

^g^ *PUCAI* paediatric ulcerative colitis activity index

^h^ *OR* odds ratio

^i^ *CI* confidence interval

^j^ *WBC* white blood cells

^k^ *HCT* haematocrit

The biochemical variables considered were the following:

- Haematocrit (%): <10 years of age (>33, 28–33, <28); 11–14 years of age/male (>35, 30–34, <30); 15–19 years of age/male (>37, 32–36, <32); 11–19 years of age/female (>37, 32–36, <32);
- Erythrocyte sedimentation rate (ESR) (mm/h): <20, 20–50, >50;
- Albumin (g/L): >35, 31–34, <30.

We also recorded Haemoglobin, mean corpuscular volume (MCV) and platelet count.

Also for the biochemical parameters, the statistical analyses aimed at comparing the two age groups were performed first on all IBD patients, and subsequently on CD patients and UC patients separately (Table 2, Additional file 1: Table S1 B).

For disease follow-up there is currently no validated clinical score that can provide a meaningful summary outcome measure. As no composite ‘damage’ score yet exists that defines overall disease burden, we still lack an accurate measure of cumulative disease burden.

In the absence of this, we considered a number of variables, referring to the main outcome measures investigated in a number of clinical studies on paediatric and adult IBD patients:

- Incremental treatment escalation included: 5-Aminosalicilates (5-ASA), exclusive enteral nutrition (EEN), antibiotics, steroids, thiopurines, biologics (Infliximab, Adalimumab), surgical intervention;
- Number of disease relapses per patient, per year of follow-up (definition of disease relapse as provided below in this paragraph);
- Number of endoscopies per patient, per year of follow-up;
- Number of unplanned inpatient and outpatient days, per patient, per year of follow-up.

As an additional investigation, Group A and B children were compared by country of origin and age group in order to explore potential differences in clinical phenotype, treatments, and disease outcome.

Disease relapse was defined as a PCDAI score [26] of above 30 for CD, or a PUCAI score [27] of above 35 for UC. Both must have persisted for at least a week despite ongoing immunosuppressant treatment. Patients with a PCDAI score between 10 and 30 or with a PUCAI score between 10 and 35 for longer than 2 weeks, and despite ongoing immunosuppressant treatment for IBD, were also considered as having a relapse. A confirmation of disease relapse by endoscopic and histological re-assessment was also available for more than 80 % of the patients enrolled in this study, but endoscopy was not considered a mandatory parameter to define a relapse of disease.

Treatment escalation in this study was defined as a) any switch from a less potent to a more potent immuno-suppressant, b) a disease relapse occurring on established maintenance therapy. The order of increasing potency was 5-ASA, oral steroids, i.v. steroids, thiopurines, biologics/surgical interventions.

Any re-induction treatment for disease relapse during the follow-up (e.g. any new course of steroids) was considered as treatment escalation.

Any induction treatment at diagnosis was not taken as treatment escalation.

An increase in dose due to weight gain was not considered as a treatment escalation, whereas an increase in dose of biologic (e.g. Infliximab, from 5 to 10 mg/Kg) or a reduction of the time between infusions (e.g., Infliximab from 8 to 6 weeks) due to loss of response, were both considered treatment escalations.

Any change in therapy made on the basis of parent/family preference, rather than on the basis of a clinical decision by the healthcare team, was not considered as treatment escalation.

### Statistical analysis

First, a descriptive statistical analysis was conducted for each clinical variable using the following tests:

- The Chi-Square test was used as a test of association for categorical variables. It allowed us to identify the existence of a correlation between a categorical variable (e.g. perianal disease at diagnosis, per rectal bleeding at diagnosis etc.) and one of the two age groups;
- For each categorical variable, a Fisher’s exact test was also used, given the small size of our cohort subsets;
- An unpaired student t-test was used to compare continuous variables (e.g. laboratory parameters) with normal distribution and similar standard deviation between the two age groups, irrespective of sample size being equal between the groups;
- Paired t-tests were used to compare the means of continuous variables between the two age groups, assuming a normal distribution but different standard deviations;
- Welch’s adaptation of Student’s t test was used when the two samples had unequal variances and unequal sample sizes;
- Mann – Whitney U test (or Wilcoxon rank-sum test) was used to compare continuous variables in the two groups, when the distribution was non-normal.

As a second step, we performed a multiple logistic regression analysis, using the SAS Statistical Package, version 9.1 (SAS Institute Inc, Cary, NC).

Multiple logistic regression analysis was performed to detect if and how independent variables (i.e. age at diagnosis, +/− country of origin) would influence disease presentation (i.e. phenotypic features at diagnoses including abdominal pain, weight loss, nocturnal symptoms, anaemia, joint pain; lab tests including albumin, CRP and ESR; disease activity scores, i.e. PCDAI and PUCAI) and disease outcomes (e.g. number of relapses per year, number of endoscopies per year, activity index at the latest follow-up (PCDAI, PUCAI), early treatment with thiopurines etc.).

We used a step-wise approach in order to weigh each single variable irrespective of the other variables (i.e. to remove inter-variable dependancy). The analysis was conducted in three main steps: first testing the variables relating to disease presentation at diagnosis (column 1 in Additional file 2: Table S2), second introducing the variables relating to disease outcomes (column 2 in Additional file 2: Table S2), finally inputting all variables (column 3 in Additional file 2: Table S2).

Variables selected for the multiple logistic regression analysis were based on the results obtained from the initial descriptive statistical analysis (comparison between the groups for each variable, through the tests described above). In fact, we selected those variables that were significantly different between the two age groups at the initial descriptive analysis, in order to investigate whether such differences would be confirmed by the multiple logistic regression analysis.

A *p* value <0.05 was considered as significant.

## Results

Patients’ demographics, including distribution by age, gender, ethnicity and type of IBD, are reported in Table 1.

We observed a higher percentage of females in the Italian Group A, which differs from what is reported in most of the literature, with usually a higher percentage of males found in the younger ages.

The vast majority of the patients enrolled in both units were Caucasians, with very little ethnic diversity (Table 1).

There was no significant difference in IBD-U frequency between the two groups in this study (22 in Group A; 17 in Group B). As this is a histological diagnosis, these results might have been expected to be skewed by the involvement of different histopathologists, however even within each centre there was no significant difference between age groups. The overall percentage of IBD-U being in line with other published results [5, 8, 10].

### Comparison of clinical phenotype at disease onset between two age groups

Children in Group A presented at diagnosis with a more severe disease activity score (PCDAI: Mean 38.75 ± 2.7; PUCAI: Mean 47.2 ± 2.5), compared to those in Group B (PCDAI: Mean 30.76 ± 2.02; PUCAI: Mean 41.36 ± 1.8) (*p* = 0.05 for CD, *p* = 0.03 for UC) (OR 1.09, 95 % CI 1.02–1.1) (Table 2a).

Using the Paris classification for IBD [28] to compare patient groups for disease location at diagnosis, we observed that an L2 location of CD was more frequent amongst the children in Group A, compared to those with later onset (*p* = 0.05) (Tables 1 and 2a). In accordance with previous studies [30], we also found that younger children had more extensive colonic involvement (*p* = 0.05), whilst the older group presented with more ileal disease.

There were no significant differences in clinical phenotype between children recruited in the UK and those recruited in Italy. Comparing clinical signs and symptoms at disease presentation, no remarkable differences emerged in predictors between the two age groups; these included gastrointestinal features (i.e. abdominal pain, diarrhoea, vomiting, PR bleeding, weight loss, faecal urgency), perianal disease and extra intestinal manifestations/associated diseases (e.g. joint involvement, erythema nodosum, sclerosing cholangitis) (Additional file 1: Table S1 A).

A more severe disease presentation amongst children in Group A was noted when children from the two countries (British and Italian) were analyzed separately. In particular, British children with CD in Group A presented more frequently with diarrhoea (*p* = 0.04), moderate-severe abdominal pain (*p* = 0.009), and urgency (*p* = 0.05), compared to those in Group B (Additional file 1: Table S1 A).

As for the Italian children, joint pain (extra intestinal manifestation of IBD) and sclerosing cholangitis (diagnosed by MRCP/liver biopsy on the basis of increased indexes of cholestasis) were observed more often at diagnosis amongst the patients in Group A than amongst those in Group B (*p* = 0.03 and *p* = 0.05 respectively) (Additional file 1: Table S1 A).

### Comparison of biochemical parameters between age groups

Overall, we did not observe any reproducible and significant differences in serum inflammatory makers (Table 2, Additional file 1: Table S1 B) between groups.

Faecal calprotectin was only available in a limited number of patients at diagnosis (four British Group A, six British Group B, 17 Italians Group A, 20 Italians Group B). When pooling all available calprotectin results at diagnosis, we found that values over 300mcg/g were present in the entire cohort, with the vast majority (45/47) having values >600 mcg/g. Over all, there were no significant differences observed between early and late onset groups, or between different centres.

Interestingly, we found a significant difference in a number of haematology indices. Children in Group A presented with higher white blood cell count (WBC) (*p* = 0.03), higher platelet count (*p* = 0.002), lower haemoglobin (*p* = 0.03) and lower haematocrit (*p* = 0.005) (Table 2b, Additional file 1: Table S1 B).

More specifically, the British children in Group A presented at diagnosis with a higher WBC count (*p* = 0.04 for all IBD, *p* = 0.02 for CD), lower hemoglobin level (*p* = 0.04 for all IBD, *p* = 0.03 for CD), lower haematocrit level (*p* = 0.02) and higher platelet count (*p* = 0.01 for all IBD – *p* = 0.001 for CD) than those in Group B (Additional file 1: Table S1 B).

Amongst Italian children, those in Group A presented at diagnosis with lower haematocrit level (*p* = 0.09, ns; *p* = 0.02 for UC), and higher platelet count (*p* = 0.003 – *p* = 0.0002 for UC), compared to those in Group B (Additional file 1: Table S1 B).

### Comparison of treatment requirements between the two groups

No differences were found between the two age groups for the number of patients treated with 5-ASA (*p* = 0.6), exclusive enteral nutrition (*p* = 0.09), antibiotics (*p* = 0.5), steroids (*p* = 0.2), biologics (*p* = 1 for Infliximab and for Adalimumab), and for the number of children who underwent surgical interventions (*p* = 0.5) (Additional file 1: Table S1 C).

**Fig. 1:**
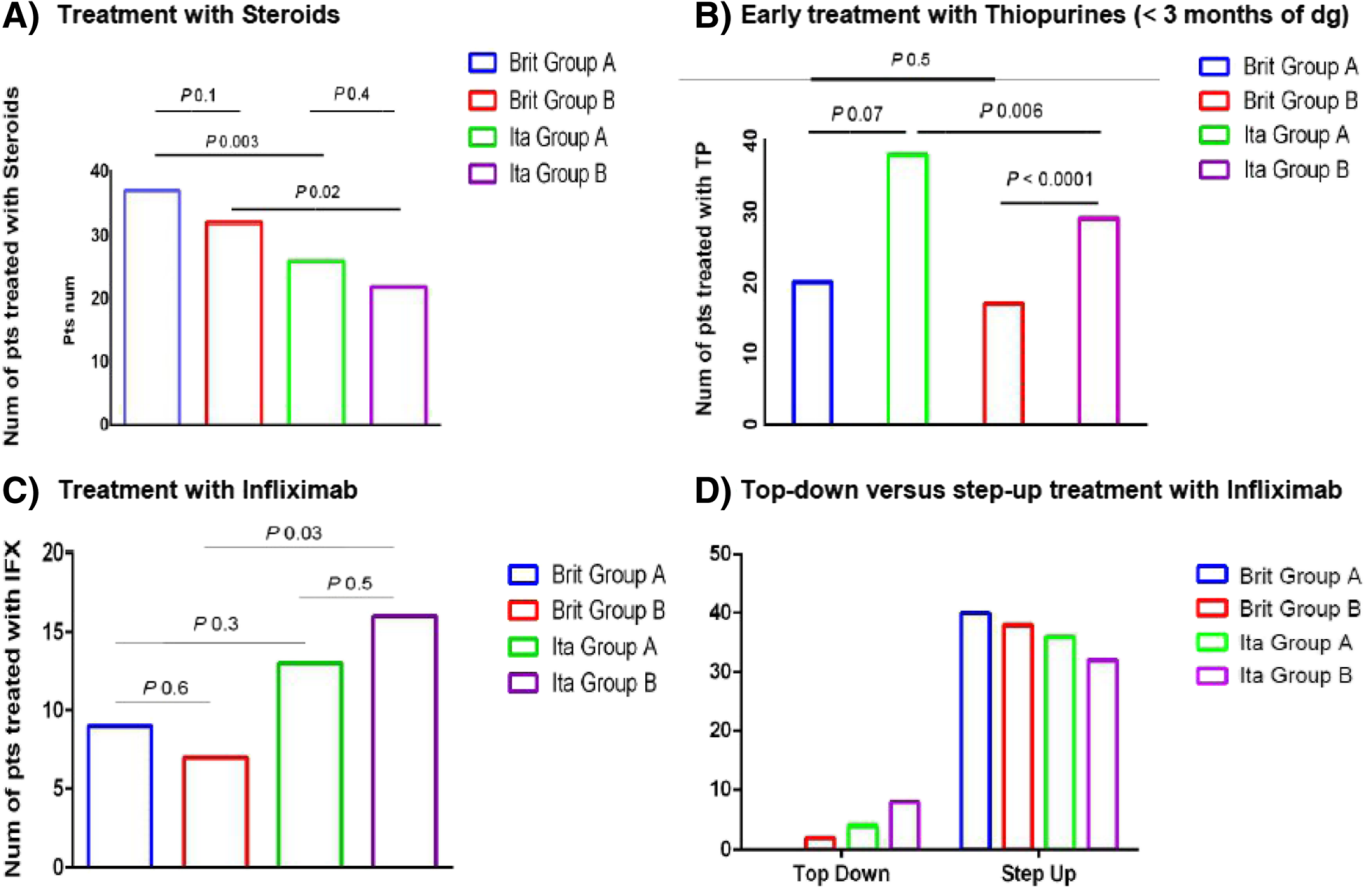
Comparison between Group A (age at diagnosis 5–10 years) and Group B (age at diagnosis ≥11 years) for treatment with Steroids (**a**), early treatment with Thiopurines (within 3 months of diagnosis) (**b**), Infliximab (**c**) and early treatment with Infliximab (top-down) (**d**). *Brit* British, *dg* diagnosis, *IFX* Infliximab, *Ita* Italian, *Num* number, *Pts* patients, *TP* Thiopurines

However, children in Group A were treated more frequently with early thiopurines (within the first 3 months after diagnosis) (*p* = 0.05) (Fig. 1b), which was also confirmed by the multiple logistic regression analysis (OR 1.86, 95 % CI 1.02–4.33; Table 2c, Additional file 2: Table S2). In particular, this was observed for the Italian children (P 0.006 for all IBD – P 0.05 for CD) (Additional file 1: Table S1 C).

No significant differences were observed between Group A and Group B, in the number of patients treated with Infliximab (*p* = 0.6 for the British and *p* = 0.5 for the Italians) (Fig. 1c and Additional file 1: Table S1 C).

Direct comparison between the cohorts from each country aimed at highlighting differences in treatment protocols between Cambridge and Padua, showing that the British patients were treated more frequently with steroids (*p* = 0.003 for Group A, *p* = 0.02 for Group B) (Fig. 1a) and with iron supplements (*p* = 0.005 for Group A) than Italian patients.

In comparison to the British, Italian children were treated more frequently with antibiotics (*p* = 0.01 for Group A, *p* = 0.006 for Group B) and with early thiopurines (i.e. within 3 months from diagnosis) (*p* <0.0001 for Group B) (OR 0.05, CI 0.01–0.21) (Fig. 1b).

When comparing British children in Group A to the Italians, no significant differences were observed in terms of treatment with Infliximab (P 0.3). However, the Italian children in Group B were treated with Infliximab more frequently than their British equivalents (*p* = 0.03) (Fig. 1c).

Moreover, Italian children also received early treatment with Infliximab (top down treatment) more frequently than the British (*p* = 0.04 both for Group A and for Group B) (Fig. 1d).

Seven children in Group A (three British, four Italians) and five in Group B (four British, one Italian) required a surgical intervention.

Overall, there were no statistically significant differences in respect to the number of surgical interventions amongst the four groups of patients (*p* = 0.7 for the British children, *p* = 0.17 for the Italian children) (Additional file 1: Table S1 C).

Amongst the three British children who had surgery in Group A, one had UC and two had IBD-U UC-like. Two of them were treated with subtotal colectomy and ileostomy, one with total colectomy and ileostomy.

Amongst the four Italian children who were operated in Group A, two had CD and two had UC. The two children with CD were treated with stricturoplasty (patient 1) and with ileal resection and drainage of abdominal abscess (patient 2).

The four British children in Group B who underwent surgery had CD. All of them were treated with laparoscopic ileo-caecal resection.

The Italian child in Group B had severe Crohn’s with fistulising perianal disease. He underwent fistulotomy and seton insertion, and was started on biologics.

### Comparison of disease outcomes between the two age groups

During follow-up, children in Group A relapsed more frequently than the ones with later onset (relapses per patient per year: mean 1.4 ± SD 0.2 for children with earlier onset vs. 0.85 ± 0.1*; p* = 0.05, OR 1.2, 95 % CI 1.01–1.65) (Table 2c).

Moreover, younger children also underwent a higher number of endoscopies (*p* = 0.04, OR 1.67, CI 1.01–4.19) (Table 2c).

Younger children had more documented relapses during their follow-up in Cambridge, whilst this group of children underwent more endoscopic investigations in Padua.

For the British patients, data on the number of unplanned inpatient and outpatient days per patient, during one year of follow-up, was also available: no significant differences were found between the two age groups (*p* = 0.2 for the number of inpatient days and *p* = 0.4 for the number of outpatient days).

## Discussion

The diagnosis of a chronic inflammatory bowel disease at any age is a major life event. It is particularly traumatic in a child, as the burden of subsequent disease is much greater, with potentially major effects on their education, growth, psychological development and long-term quality of life. It is clear that children diagnosed with IBD under the age of five represent a unique disease group that is different to the more ‘typical’ IBD phenotype. These are often caused by specific genetic variations and/or rare abnormalities in immune defence [13–18]. However, after the age of about 5 years, it is uncertain if age itself remains a further predictor of disease course and outcome.

To explore this hypothesis, we provide a detailed analysis of 160 children with IBD recruited in two large tertiary European centres. We compared the lower quartile of children aged 5–10 years with those aged 11–16 years, seeking to identify clinically useful differences between these two age groups.

The most significant distinction between age-groups was that disease activity scores at diagnosis (PCDAI, PUCAI) were higher amongst the younger group of children (*p* = 0.05 for CD, *p* = 0.03 for UC). However these scores are all based on retrospective calculation of disease activity, something that may compromise the validity of the statistical significance.

In addition, we found only minor differences on multiple logistic regression analysis (OR 1.09, 95 % CI 1.02–1.1), suggesting further caution is required before claiming clinically relevant differences at diagnosis.

We acknowledge that recruiting patients from two different countries might introduce a significant geographical bias. As an example, disease activity at diagnosis was more severe in the British Group A than in the older group, whereas such difference was not significant amongst the Italian children (Table 2). A difference in disease activity at diagnosis was also observed once all children are pooled together and the two age groups were compared, which is likely to be driven by the British cohorts. In order to reduce biases in the downstream analysis (e.g., treatment protocols and outcomes compared between two populations of children who may have differences related to their country of origin), the statistical analysis for each variable was performed in two steps: first comparing the two age groups within each country, and subsequently pooling all children from the different countries together, to compare the two age categories on higher numbers. The multiple logistic regression analysis was also performed twice, first considering and then disregarding the variable “country of origin”. This hasn’t really reflected on any other significant difference in terms of clinical phenotype, laboratory parameters and outcomes between the two age groups. Moreover, the primary aim of this study was to detect specific phenotypic and outcome differences in children diagnosed with IBD in different age groups, and we consider that any consistent differences age-related should have emerged in our study, irrespective of the country of origin.

Some of the variables we analysed were objective parameters collected reliably from records held at both centres (e.g. clinical signs and symptoms at diagnosis, laboratory results, number of surgeries etc.). A specific comment is required in regard to joint involvement as extra-intestinal manifestation (EIM) at disease presentation, which should be interpreted carefully, in view of previous studies investigating the same parameter [29]. In fact, although our study was led by a single researcher working in both centres, it was still a retrospective data collection and analysis, meaning different clinicians assessed the EIMs.

Although both units started children in the younger age group at diagnosis on immunosuppressants more frequently in the first 3 months (95 % of Italian Group-A and 50 % of British Group-A), Italian adolescents were given Infliximab sooner (15 % vs 2.5 %) and more frequently (36.2 % vs 16.3 %) than their British counterparts.

Parameters like type of medication, number of treatment escalations per patient per year, endoscopies per patient per year, etc. are all likely to be influenced by local practice. Although this variability remains an intrinsic limitation of this study, it suggests clear variation in treatment in the context of little difference in clinical outcome. As outlined in the methods section, we did do our best to standardise data collection as far as possible.

Our findings clearly demonstrate different clinical approaches to treatment with immunomodulators and biologics in each unit. The UK Unit typically employed a “step-up” approach to treatment, reserving biologic treatment as third line therapy, whilst the Italian Unit showed a clear preference for a “top-down” strategy. Both centres have unrestricted access to biological therapy, making clinical indications the main drivers for use. However, it is well known that Italian paediatric gastroenterology training and principals are more aligned with US practice than with the European ‘step-up’ approach.

It is very telling therefore, that we identified only modest differences in clinical outcome between children treated with a ‘top-down’ rather than ‘step-up’ approach. However, our findings do suggest that younger children present with more severe disease, relapse more frequently and are endoscoped more frequently than their older counterparts – all despite earlier treatment with both immunosuppressants (UK and Italy) and biologics (Italy only). Furthermore the higher relapse rate in the younger age group in the UK, should be seen in the context of more steroid use and later intervention with biologics.

On this basis, the question remains whether a more aggressive treatment is still really justified in children with disease onset between 5 and 10 years of age?

## Conclusion

In keeping with previous evidence on this topic, our findings confirm a more extensive disease location and a greater disease activity at presentation, in children diagnosed with IBD between the ages of 5 and 10 years, compared to adolescents. Whilst the numbers in this retrospective study are relatively small, our analysis also suggests a greater disease burden in the younger children (e.g. higher number of relapses and endoscopies). However, randomised trials to document longer-term clinical outcomes are urgently needed, in order to address the question whether a younger age at disease onset should prompt *per se* a more “aggressive” treatment (e.g. early immunosuppression, “top-down” use of biologics).

Given the expanding research availabilities in IBD, we speculate that new approaches (e.g. epigenetics, development of new prognostic biomarkers) may be more helpful than simple clinical parameters, in order to predict outcomes in different patient groups, to individualise our decision-making and to personalise treatment at diagnosis.

## Additional files

Additional file 1: Table S1. Main clinical features at diagnosis. Comparison between children with earlier onset of IBD (Group A, 5 to 10 years of age at diagnosis) and later onset (Group B, 11 to 16 years of age at diagnosis). (DOC 24 kb) Additional file 2: Table S2. Multiple logistic regression analysis comparing Group A (age at diagnosis 5–10 years) and Group B (age at diagnosis ≥11 years). A) Analysis conducted irrespective of the country of origin. B) Analysis conducted considering the country of origin (United Kingdom, Italy). (PDF 82 kb)

## Abbreviations

CD: Crohn’s diseases
EEN: exclusive enteral nutrition
ESR: erythrocyte sedimentation rate
IBD: inflammatory bowel disease
IBD-U: inflamamtory bowel disease unclassified
MCV: mean corpuscular volume
PCDAI: paediatric Crohn’s disease activity index
PUCAI: paediatric ulcerative colitis activity index
UC: ulcerative colitis
WBC: white blood cells
5-ASA: 5 amino salicylates
