# Supplementary material for: Clinical course and outcomes of diagnosing Inflammatory Bowel Disease in children 10 years and under: retrospective cohort study from two tertiary centres in the United Kingdom and in Italy
Source: BMC Gastroenterol. 2016 Mar 15;16:35. doi: 10.1186/s12876-016-0455-y (PMC4791934; doi:10.1186/s12876-016-0455-y)
Supplement: Additional file 1: Table S1. — Main clinical features at diagnosis. Comparison between children with earlier onset of IBD (Group A, 5 to 10 years of age at diagnosis) and later onset (Group B, 11 to 16 years of age at diagnosis). (DOC 24 kb) [file 12876_2016_455_MOESM1_ESM.doc]

**Supplementary Table S1. Main clinical features at diagnosis. Comparison between children with earlier onset of IBD (Group A, 5 to 10 years of age at diagnosis) and later onset (Group B, 11 to 16 years of age at diagnosis).**

**A) Symptoms and signs at disease presentation**

| **Symptoms / Signs** | **All Group A vs Group B** | **Brit[[1]](#footnote-2) Group A vs Group B** | **Ita[[2]](#footnote-3) Group A vs Group B** | **Brit1 CD[[3]](#footnote-4) Group A vs Group B** | **Brit1 UC[[4]](#footnote-5) Group A vs Group B** | **Ita2 CD Group A vs Group B** | **Ita2 UC Group A vs Group B** |
| --- | --- | --- | --- | --- | --- | --- | --- |
| **Diarrhoea**  **Group A**  **Group B**  ***P*** | 69  60  *0.07* | 37  33  *0.17* | 32  27  *0.2* | 18 / 19  17 / 24  ***0.04*** | 9 / 21  16 / 16  *0.2* | 13 / 17  19 / 23  *0.07* | 11 / 23  16 / 17  *0.3* |
| **Urgency**  **Group A**  **Group B**  ***P*** | 53  44  *0.15* | 31  20  ***0.01*** | 22  24  *0.6* | 13 / 19  11 / 24  ***0.05*** | 18 / 21  9 / 16  ***0.05*** | 6 / 17  9 / 23  *0.8* | 16 / 23  15 / 17  *0.2* |
| **Low Energy levels**  **Group A**  **Group B**  ***P*** | 41  42  *0.9* | 24  27  *0.5* | 17  15  *0.6* | 14 / 19  18 / 24  *0.9* | 10 / 21  9 / 16  *0.6* | 9 / 17  12 / 23  *0.9* | 8 / 23  3 / 17  *0.2* |
| **Nocturnal symptoms**  **Group A**  **Group B**  ***P***  **OR[[5]](#footnote-6)**  **95% CI[[6]](#footnote-7)** | 33  80  *0.5*  **1.54**  0.62 - 3.85 | 27  30  *0.5*  **4.13**  **1 - 19.1** | 6  7  *0.8*  0.97  0.12 - 7.68 | 13 / 19  19/ 24  *0.4* | 14 / 21  11 / 16  *0.9* | 3 / 17  3 / 23  *0.7* | 3 / 23  4 / 17  0.4 |
| **Abdominal pain**  **Group A**  **Group B**  ***P***  **OR**  **95% CI** | 71  69  *0.8*  **1.31**  0.4 - 4.3 | 40  36  ***0.04***  **1.38**  0.41 - 4.59 | 31  33  *0.2*  0.92  0.16 - 5.13 | 19 / 19  12 / 24  ***0.009*** | 21 / 21  14 / 16  *0.6* | 14 / 17  20 / 23  *0.7* | 17 / 23  13 / 17  *0.8* |
| **Joint pain**  **Group A**  **Group B**  ***P***  **OR**  **95% CI** | 13  16  *0.5*  **1.41**  0.58 - 3.42 | 12  9  *0.5*  **3.19**  0.68 - 15.04 | 7  1  ***0.03***  **1.37**  0.24 - 8.02 | 7 / 19  5 / 24  *0.2* | 5 / 21  4 / 16  *0.9* | 6 / 17  0 / 23  ***0.02*** | 1 / 23  1 / 17  *0.8* |
| **PR[[7]](#footnote-8) bleeding**  **Group A**  **Group B**  ***P*** | 63  56  *0.2* | 35  28  *0.06* | 28  28  *1* | 15 / 19  14 / 24  *0.3* | 21 / 21  14 / 16  *0.1* | 7 / 17  11 / 23  *0.7* | 21 / 23  17 / 17  *0.2* |
| **Wt[[8]](#footnote-9) loss**  **Group A**  **Group B**  ***P***  **OR**  **95% CI** | 36  46  *0.25*  **0.65**  0.3 - 1.41 | 22  27  *0.3*  **0.15**  **0.031 - 0.692** | 14  19  *0.9*  **1.5**  0.39 - 5.8 | 14 / 19  18 / 24  *0.5* | 8 / 21  9 / 16  *0.3* | 7 / 17  15 / 23  *0.1* | 7 / 23  4 / 17  *0.6* |
| **Vomit**  **Group A**  **Group B**  ***P*** | 8  14  *0.2* | 7  14  *0.08* | 1  0  *0.3* | 6 / 19  11 / 24  *0.3* | 1 / 21  3 / 16  *0.2* | 0 / 17  0 / 23  *1* | 1 / 23  0 / 17  *0.4* |
| **Abdominal tenderness**  **Group A**  **Group B**  ***P*** | 27  28  *0.9* | 15  21  *0.2* | 12  7  *0.2* | 9 / 19  16 / 24  *0.2* | 6 / 21  5 / 16  *0.9* | 6 / 17  5 / 23  *0.3* | 6 / 23  2 / 17  *0.4* |
| **Abdominal masses**  **Group A**  **Group B**  ***P*** | 68  68  *1* | 15  21  *0.2* | 12  7  *0.4* | 9 / 19  16 / 24  *0.09* | 6 / 21  5 / 16  *0.9* | 6 / 17  5 / 23  *0.9* | 6 / 23  2 / 17  *0.2* |
| **Mouth ulcers**  **Group A**  **Group B**  ***P*** | 7  11  *0.7* | n.a.  n.a.  n.a. | n.a.  n.a.  n.a. | 5 / 19  10 / 24  *0.3* | n.a.  n.a.  n.a. | 2 / 17  1 / 23  *0.4* | n.a.  n.a.  n.a. |
| **Perianal disease**  **CD**  **Group A**  **Group B**  ***P*** | 11  14  *0.9* | n.a.  n.a.  n.a. | n.a.  n.a.  n.a. | 6 / 19  6 / 24  *0.6* | n.a.  n.a.  n.a. | 5 / 17  8 / 23  *0.7* | n.a.  n.a.  n.a. |
| **Fever**  **Group A**  **Group B**  ***P*** | 17  14  *0.5* | 5  *7*  *0.4* | 12  10  *0.6* | 3 / 19  4 / 24  *0.9* | 2 / 21  0 / 16  *0.2* | 4 / 17  8 / 23  *0.4* | 8 / 23  2 / 17  *0.1* |
| **Anaemia**  **Group A**  **Group B**  ***P*** | 37  28  **0.02** | 23  20  *0.5* | 14  8  *0.1* | 14 / 19  13 / 24  *0.2* | 9 / 21  7 / 16  *0.9* | 3 / 17  3 / 23  *0.7* | 11 / 23  5 / 17  *0.2* |
| **Sclerosing cholangitis**  **Group A**  **Group B**  ***P*** | 70  75  *0.18* | 4  4  *1* | 6  1  ***0.05*** | 0 / 19  1 / 24  *0.4* | 4 / 21  3 / 16  *0.9* | 1 / 17  1 / 23  *0.8* | 5 / 23  0 / 17  ***0.04*** |

**B) Laboratory parameters at diagnosis.**

| **Laboratory parameters** | **All Group A vs Group B** | **Brit Group A vs Group B** | **Ita Group A vs Group B** | **Brit CD Group A vs Group B** | **Brit UC Group A vs Group B** | **Ita CD Group A vs Group B** | **Ita UC Group A vs Group B** |
| --- | --- | --- | --- | --- | --- | --- | --- |
| **WBC [[9]](#footnote-10)**  **(x10^3/mm3)**  **Group A**  mean ± SD[[10]](#footnote-11)  median  range  **Group B**  mean ± SD  median  range  ***P*** | 10.1 ± 0.45  9.53  7.6-20.7  8.81 ± 0.35  8.53  3.7 - 19  ***0.03*** | 10.9 ± 0.7  10.4  4.5 – 20.7  9.09 ± 0.5  8.4  3.7 – 19  ***0.04*** | 9.4 ± 0.6  8.9  3.4 – 19.4  8.5 ± 0.47  8.6  3.9 – 18.9  *0.3* | 12.04 ± 1.1  11.9  4.5 – 20.7  9.09 ± 0.5  8.9  4.8 – 14  ***0.02*** | 9.7 ± 0.8  9.1  4.8 – 17.2  9.09 ± 1.1  7.5  3.7 – 19  *0.7* | 7.9 ± 0.5  7.6  3.4 – 11.2  8.7 ± 0.5 8.8  4.5 – 13.2  *0.2* | 10.5 ± 0.9  10.2  5.2 – 19.4    8.2 ± 0.9  7.8  3.8 – 18.9  *0.08* |
| **Hemoglobin**  **(g/dL)**  **Group A**  mean ± SD  median  range  **Group B**  mean ± SD  median  range  ***P*** | 11.01 ± 0.2  11  5.8 - 15.4  11.5 ± 0.2  11.6  6.2 - 15.8  **0.03** | 10.6 ± 0.23  10.6  7.4 – 14.4  11.4 ± 0.26  11.4  6.5 – 14  **0.04** | 11.4 ± 0.34  11.7  5.8 – 15.4  11.8 ± 0.29  11.8  6.2 – 15.8  0.4 | 10.4 ± 0.25  10.6  7.4 – 11.8  11.3 ± 0.3  11.5  7.6 – 13.8  **0.03** | 10.9 ± 0.4  11.1  8.2 – 14.4  11.4 ± 0.5  11.4  6.5 – 14  0.4 | 11.9 ± 0.4  12.1  7.4 – 15.4  11.5 ± 0.3  11.3  8.5 – 15.8  0.5 | 11 ± 0.5  10.8  5.8 – 15.3  12 ± 0.5  12.4  6.2 – 15.1  0.2 |
| **MCV [[11]](#footnote-12)(fL)**  **Group A**  mean ± SD  median  range  **Group B**  mean ± SD  median  range  ***P*** | 76.04 ± 0.9  77.5  52 - 87.9  77.6 ± 0.8  68  71 - 95.1  *0.2* | 76.1 ± 1.2  74.9  62.2 – 87.9  77.9 ± 1.1  77.9  61 – 95.1  *0.2* | 76 ± 1.2  78  52 – 87  77.4 ± 1.2  78  64 – 88  *0.4* | 74.3 ± 1.4  73.1  63.4 – 85.1  75.2 ± 1.5  75.9  61 – 95.1  *0.7* | 78 ± 1.8  80.5  62.2 – 87.9  81.8 ± 1.1  81.7  74 – 90  *0.09* | 77.5 ± 1.6  79  66 – 87  74.7 ± 1.4  75  64 – 86  *0.2* | 74.8 ± 1.6  78  52 – 85  81.3 ± 1.4  83  68 – 88  ***0.008*** |
| **Hct [[12]](#footnote-13)(%)**  **Group A**  mean ± SD  median  range  **Group B**  mean ± SD  median  range  ***P*** | 33.7 ± 0.57  34.3  20 - 44.9  35.8 ± 0.5  35.9  19.6 - 47.6  ***0.005*** | 32.6 ± 0.6  32.8  24.7 – 41.7  34.9 ± 0.7  35.1  19.6 – 41.7  ***0.02*** | 34.8 ± 0.9  35.5  20 – 44.9  36.7 ± 0.6  36.7  28 – 47.6  *0.09* | 32.3 ± 0.7  32.8  24.7 – 36.2  34.8 ± 0.7  34.6  25 – 41.7  ***0.02*** | 32.9 ± 1.06  34  26.1 – 41.7  33.8 ± 1.5  34.6  19.6 – 41.7  *0.6* | 36.3 ± 0.9  36.6  25.9 – 44.9  36.1 ± 0.9  35.8  28 – 47.6  *0.9* | 33.6 ± 1.4  34.2  20 – 43.6  37.6 ± 0.9  37.3  31.4 – 44  ***0.02*** |
| **Platelet count**  **(x10^3/mm3)**  **Group A**  mean ± SD  median  range  **Group B**  mean ± SD  median  range  ***P*** | 487 ± 18.9  456  196 - 968  393 ± 13.4  380  201 - 793  **0.002** | 476 ± 21.3  493  196 – 796  404 ± 18.6  403  234 – 793  **0.01** | 497.5 ± 31.2  438  196 – 968  382 ± 19.3  364  201 – 762  **0.003** | 539 ± 24.1  534  369 – 796  420 ± 24.5  421  234 – 793  **0.001** | 410.8 ± 28.9 414.5  196 – 609  382 ± 28.5  355  242 – 633  0.5 | 471 ± 36.8  428  318 – 853  442 ± 23.7  432  232 – 762  0.5 | 518 ± 47.7  448  196 – 968  295.4 ± 16.6 301  201 – 405  **0.0002** |

**C) Treatments**

| **Treatments** | **All Group A vs Group B** | **Brit1 Group A vs Group B** | **Ita2 Group A vs Group B** | **Brit1 CD3 Group A vs Group B** | **Brit1 UC4 Group A vs Group B** | **Ita2 CD3 Group A vs Group B** | **Ita2 UC4 Group A vs Group B** |
| --- | --- | --- | --- | --- | --- | --- | --- |
| **5 - ASA[[13]](#footnote-14)**  **Group A**  **Group B**  ***P*** | 69 / 80  66 / 80  *0.6* | 34 / 40  34 / 40  *1* | 35 / 40  32 / 40  *0.9* | 14 / 19  19 / 24  *0.7* | 20 / 21  15 / 16  *0.8* | 15 / 17  15 / 23  *0.9* | 20 / 23  17 / 17  *0.2* |
| **Antibiotics**  **Group A**  **Group B**  ***P*** | 35 / 80  30 / 80  *0.5* | 12 / 40  9 / 40  *0.4* | 23 / 40  21 / 40  *0.7* | 5 / 19  6 / 24  *0.9* | 7 / 21  3 / 16  *0.3* | 8 / 17  13 / 23  *0.6* | 15 / 23  8 / 17  *0.25* |
| **EEN[[14]](#footnote-15)**  **CD**  **Group A**  **Group B**  ***P*** | 21 / 80  31 / 80  *0.09* | n.a.  n.a.  n.a. | n.a.  n.a.  n.a. | 9 / 19  12 / 24  *0.4* | n.a.  n.a.  n.a. | 12 / 17  19 / 23  *0.1* | n.a.  n.a.  n.a. |
| **Steroids**  **Group A**  **Group B**  ***P*** | 63 / 80  54 / 80  *0.2* | 37 / 40  32 / 40  *0.1* | 26 / 40  22 / 40  *0.4* | 16 / 19  18 / 24  *0.5* | 21 / 21  14 / 16  *0.1* | 10 / 17  8 / 23  *0.2* | 16 / 23  14 / 17  *0.4* |
| **Thiopurines**  **Group A**  **Group B**  ***P*** | 63 / 80  61 / 80  *0.8* | 33 / 40  26 / 40  *0.15* | 38 / 40  35 / 40  *0.2* | 17 / 19  19 / 24  *0.4* | 16 / 21  7 / 16  ***0.04*** | 17 / 17  20 / 23  *0.7* | 21 / 23  15 / 17  *0.9* |
| **Early Thiopurines**  **Group A**  **Group B**  ***P***  **OR**  **95% CI** | 58 / 80  46 / 80  ***0.05***  **1.86**  1.02 - 4.33 | 20 / 40  17 / 40  *0.7*  **1.05**  0.68 - 2.63 | 38 / 40  19 / 40  ***0.006***  **7.19**  0.78 - 77.4 | 14 / 19  12 / 24  *0.8* | 6 / 21  5 / 16  *0.9* | 17 / 17  9 / 23  ***0.05*** | 21 / 23  10 / 17  *0.1* |
| **Infliximab**  **Group A**  **Group B**  ***P*** | 22 / 80  23 / 80  *1* | 9 / 40  7 / 40  *0.6* | 13 / 40  16 / 40  *0.5* | 7 / 19  5 / 24  *0.2* | 2 / 21  2 / 16  *0.7* | 11 / 17  15 / 23  *0.9* | 2 / 23  1 / 17  *1* |
| **Adalimumab**  **Group A**  **Group B**  ***P*** | 6 / 80  6 / 80  *1* | 1 / 40  2 / 40  *0.6* | 5 / 40  4 / 40  *0.7* | 1 / 19  2 / 24  *0.7* | 0 / 21  0 / 16  *1* | 5 / 17  4 / 23  *0.4* | 0 / 23  0 / 17  *1* |
| **Early biologics (top-down)**  **Group A**  **Group B**  ***P*** | 4 / 80  10 / 80  0.1 | 0 / 40  2 / 40  *0.15* | 4 / 40  8 / 40  *0.2* | 0 / 19  1 / 24  *0.4* | 0 / 21  1 / 16  *0.2* | 2 / 17  7 / 23  *0.2* | 2 / 23  1 / 17  *0.7* |
| **Surgical interventions**  **Group A**  **Group B**  ***P*** | 7 / 80  5 / 80  0.5 | 3 / 40  4 / 40  0.7 | 4 / 40  1 / 40  0.17 | 0 / 19  4 / 24  0.06 | 3 / 21  0 / 16  0.14 | 2 / 17  1 / 23  0.4 | 2 / 23  0 / 17  0.2 |

1. Brit: British [↑](#footnote-ref-2)
2. Ita: Italian [↑](#footnote-ref-3)
3. CD: Crohn's Disease [↑](#footnote-ref-4)
4. UC: Ulcerative Colitis [↑](#footnote-ref-5)
5. OR: Odds ratio [↑](#footnote-ref-6)
6. CI: Confidence Interval [↑](#footnote-ref-7)
7. PR: Per rectum [↑](#footnote-ref-8)
8. Weight [↑](#footnote-ref-9)
9. WBC: White blood cells [↑](#footnote-ref-10)
10. SD: Standard Deviation [↑](#footnote-ref-11)
11. MCV: Mean Cell Volume [↑](#footnote-ref-12)
12. Hct: Haematocrit [↑](#footnote-ref-13)
13. 5-ASA: 5-Aminosalicilates (Mesalazine, Sulphasalazine) [↑](#footnote-ref-14)
14. EEN: Exclusive enteral nutrition [↑](#footnote-ref-15)
