# Supplementary material for: Clinical course and outcomes of diagnosing Inflammatory Bowel Disease in children 10 years and under: retrospective cohort study from two tertiary centres in the United Kingdom and in Italy
Source: BMC Gastroenterol. 2016 Mar 15;16:35. doi: 10.1186/s12876-016-0455-y (PMC4791934; doi:10.1186/s12876-016-0455-y)
Supplement: Additional file 2: Table S2. — Multiple logistic regression analysis comparing Group A (age at diagnosis 5–10 years) and Group B (age at diagnosis ≥11 years). A) Analysis conducted irrespective of the country of origin. B) Analysis conducted considering the country of origin (United Kingdom, Italy). (PDF 82 kb) [file 12876_2016_455_MOESM2_ESM.pdf]

Supplementary Table S2. Multiple logistic regression analysis comparing Group A (age at diagnosis 5-10 years) and Group B (age at diagnosis ≥ 11 years). A) Analysis conducted irrespective of the country of origin. B) Analysis conducted considering the country of origin (United Kingdom, Italy).

A)

		Disease phenotype at dg[1]			Disease outcomes			All variables		
		OR[2]	CI[3] 95%		OR	CI 95%		OR	CI 95%	
Diagnosis	CD[4] + IBD-U[5] CD-like	ref.[6]			ref.			ref.		
	UC[7] + IBD-U UC-like	0.467	0.192	1.138	1.190	0.614	2.306	0.553	0.217	1.410
Time onset - dg (yrs[8])		0.923	0.568	1.501				0.942	0.561	1.583
Moderate to severe abdo[9] pain at dg	NO	ref.						ref.		
	YES	1.026	0.326	3.234				1.307	0.399	4.283
Wt[10] loss at dg	NO	ref.						ref.		
	YES	0.686	0.325	1.449				0.647	0.296	1.412
Nocturnal symptoms at dg	NO	ref.						ref.		
	YES	1.607	0.677	3.812				1.542	0.618	3.846
Anemia at dg	NO	ref.						ref.		
	YES	0.768	0.363	1.623				0.825	0.377	1.802
Joint pain at dg	NO	ref.						ref.		
	YES	1.450	0.616	3.413				1.411	0.582	3.420
Albumin at dg		1.034	0.964	1.110				1.027	0.952	1.107
CRP[11] at dg		0.996	0.979	1.013				0.991	0.972	1.009
ESR[12] at dg		1.005	0.989	1.021				1.004	0.987	1.021
**PCDAI[13]/PUCAI[14] at dg**		**1.040**	**1.008**	**1.074**				**1.044**	**1.010**	**1.080**
PCDAI/PUCAI at follow-up					0.975	0.945	1.007	0.967	0.932	1.003
Relapses / pt[15] / yr					1.139	0.764	1.697	1.045	0.875	1.619
Endoscopies / pt / yr					1.493	0.712	3.130	1.674	0.769	4.188
Early treatment with Thiopurines	NO				ref.			ref.		
	YES				1.341	0.672	2.677	1.862	0.900	4.332

B)

		Disease phenotype at dg			Disease outcomes			All variables		
		OR	CI 95%		OR	CI 95%		OR	CI 95%	
Diagnosis	CD + IBD-U CD-like	ref.			ref.			ref.		
	UC + IBD-U UC-like	0.457	0.186	1.123	1.179	0.606	2.294	0.550	0.214	1.412
Time onset - dg (yrs)		0.922	0.565	1.504				0.927	0.547	1.569
Moderate to severe abdo pain	NO	ref.						ref.		
	YES	1.100	0.341	3.546				1.378	0.413	4.599
Wt loss	NO	ref.						ref.		
	YES	0.707	0.333	1.500				0.670	0.305	1.473
Nocturnal symptoms	NO	ref.						ref.		
	YES	1.786	0.734	4.349				1.725	0.674	4.415
Anemia	NO	ref.						ref.		
	YES	0.845	0.391	1.826				0.943	0.419	2.124
Joint pain	NO	ref.						ref.		
	YES	1.564	0.652	3.752				1.498	0.611	3.673
Albumin		1.026	0.955	1.103				1.014	0.937	1.096
CRP		0.996	0.979	1.014				0.992	0.973	1.011
ESR		1.002	0.985	1.019				1.001	0.983	1.019
**PCDAI/PUCAI at dg**		**1.041**	**1.009**	**1.075**				**1.090**	**1.020**	**1.100**
PCDAI/PUCAI at follow-up					0.975	0.944	1.006	0.963	0.928	1.000
**Relapses / pt / yr**					1.144	0.864	1.714	**1.200**	**1.010**	**1.650**
**Endoscopies / pt / yr**					1.502	0.814	3.158	**1.670**	**1.015**	**4.190**
**Early treatment with Thiopurines**	NO				ref.			ref.		
	YES				1.341	0.872	2.774	**1.862**	**1.020**	**4.330**
Country	Great Britain	0.626	0.266	1.473	0.909	0.442	1.870	0.535	0.196	1.456
	Italy	ref.			ref.			ref.		

[1] dg: diagnosis;
[2] OR: Odds ratio;
[3] CI: Confidence Interval;
[4] CD: Crohn's disease;
[5] IBD-U: Inflamamtory Bowel Disease Unclassified;
[6] ref.: reference;
[7] UC: ulcerative colitis;
[8] yr(s): year(s);
[9] abdo: abdominal;
[10] Wt: weight;
[11] CRP: C-reactive protein;
[12] ESR: erythrocyte sedimentation rate;
[13] PCDAI: Paediatric Crohn's Disease Activity Index;
[14] PUCAI: Paediatric Ulcerative Colitis Activity Index;
[15] pt: patient.
